# Supplementary material for: Synthesis of New 1,4-Naphthoquinone Fluorosulfate Derivatives and the Study of Their Biological and Electrochemical Properties
Source: Int J Mol Sci. 2024 Nov 14;25(22):12245. doi: 10.3390/ijms252212245 (PMC11595164; doi:10.3390/ijms252212245)
Supplement: Supplementary file 1 [file ijms-25-12245-s001.zip › ijms-3284153-supplementary.pdf]

Synthesis of new 1,4-naphthoquinone fluorosulfate detivatives and the study of their biological and electrochemical properties

Natalia V. Aseeva [1,*], Nadezhda V. Danilenko [2], Evgenii V. Plotnikov [3], Elena I. Korotkova [1], Olga I. Lipskikh [1], Anna N. Solomonenko [1], Alina V. Erkovich [1], Daria D. Eskova [3], Andrei I. Khlebnikov [2,*]

[1] Engineering School of Natural Resources, Department of Chemical Engineering, National Research Tomsk Polytechnic University, Lenin Avenue, 30, Tomsk, 634034, Russian Federation; natali.shkuratova@mail.ru (N.V.A.), eikor@mail.ru (E.I.K), lipskih-olga@yandex.ru (O.I.L.); ans51@tpu.ru (A.N.S.); avg48@tpu.ru (A.V.E.);

[2] The School of Advanced Manufacturing Technologies, National Research Tomsk Polytechnic University, Lenin Avenue, 30, Tomsk, 634034, Russian Federation; aikhl@chem.org.ru (A.I.K.); nadezhda.dani@gmail.com (N.V.D.);

[3] Research School of Chemistry & Applied Biomedical Sciences, National Research Tomsk Polytechnic University, Lenin Avenue, 30, Tomsk, 634034, Russian Federation; plotnikov.e@mail.ru (E.V.P.); dde5@tpu.ru (D.D.E.)

Supplementary information

Supplementary data: The view of the two-chamber reactor; the ^{1}H, ^{13}C, ^{19}F NMR spectra; PASS prediction of biological activities for the naphthoquinone derivatives; results of the DFT calculations.

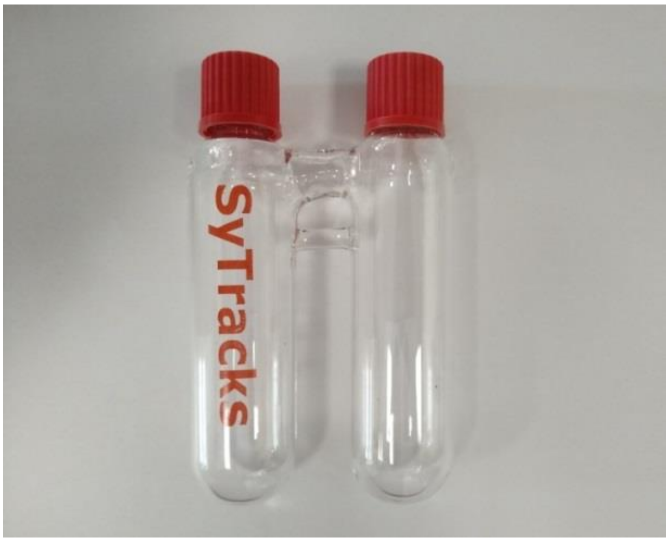

Figure S1. The two-chamber reactor

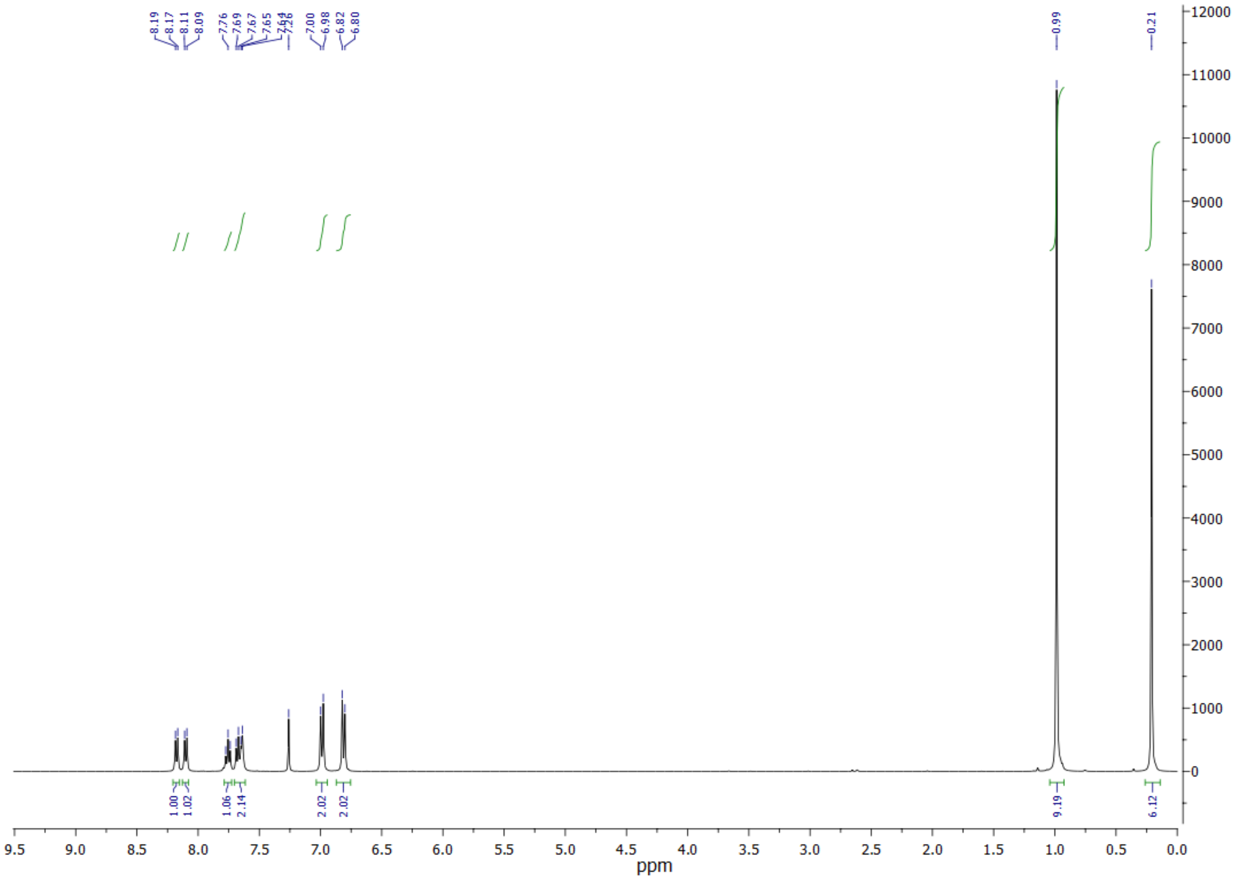

Figure S2. ^{1}H NMR spectrum of compound **NQ1a** (400 MHz, $CDCl_3$)

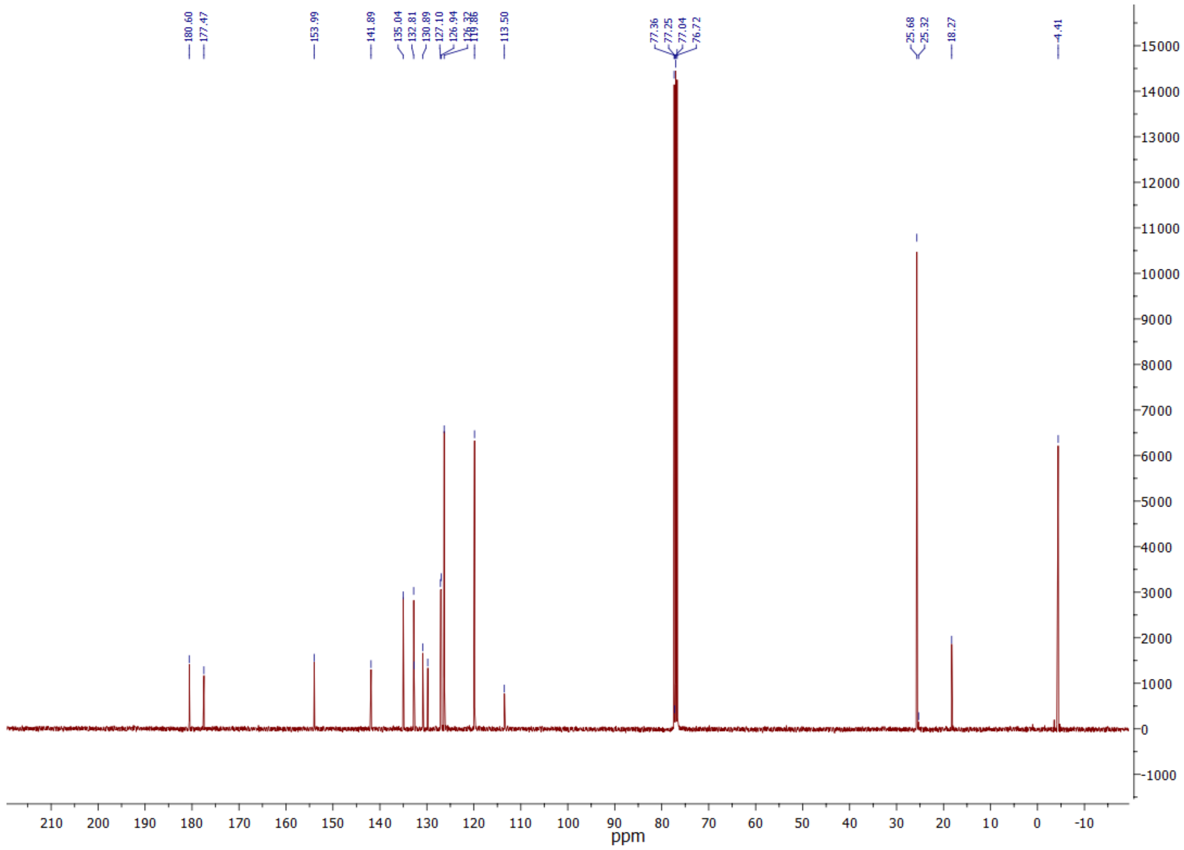

Figure S3. ^{13}C NMR spectrum of compound **NQ1a** (100 MHz, $CDCl_3$)

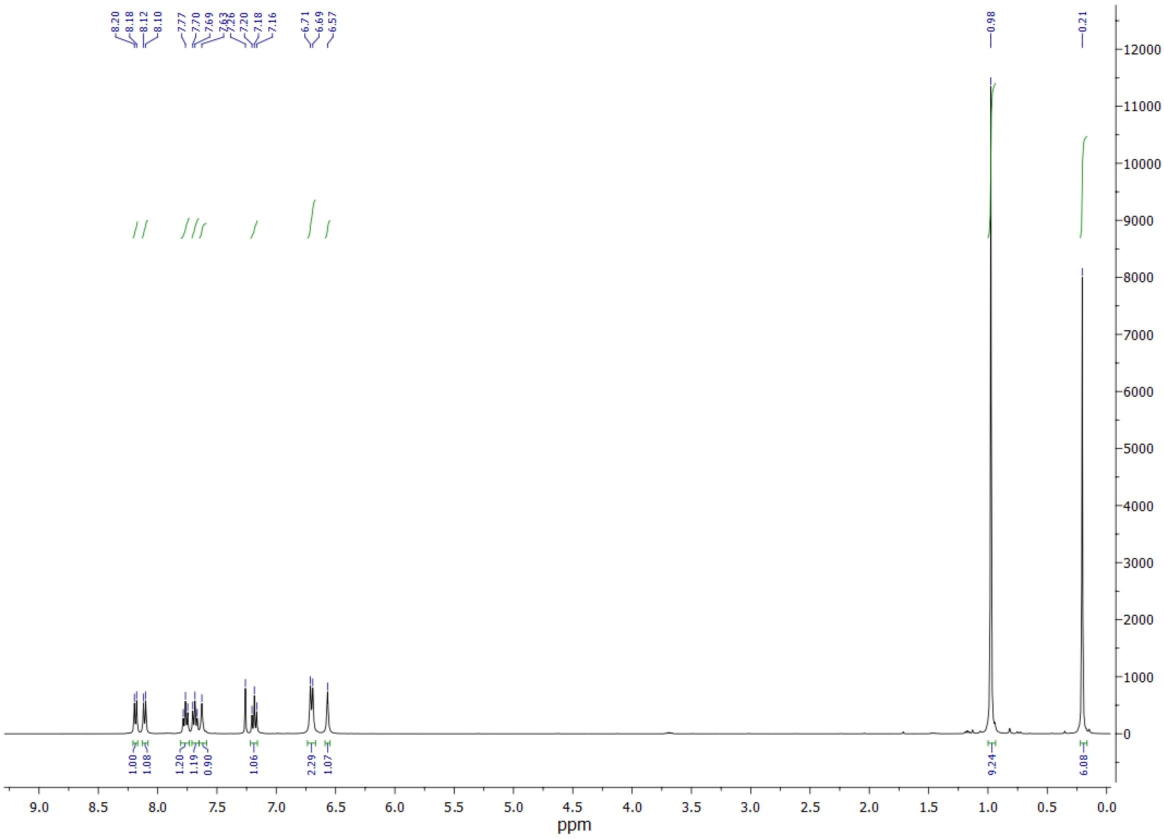

Figure S4. ^{1}H NMR spectrum of compound **NQ2a** (400 MHz, $CDCl_3$)

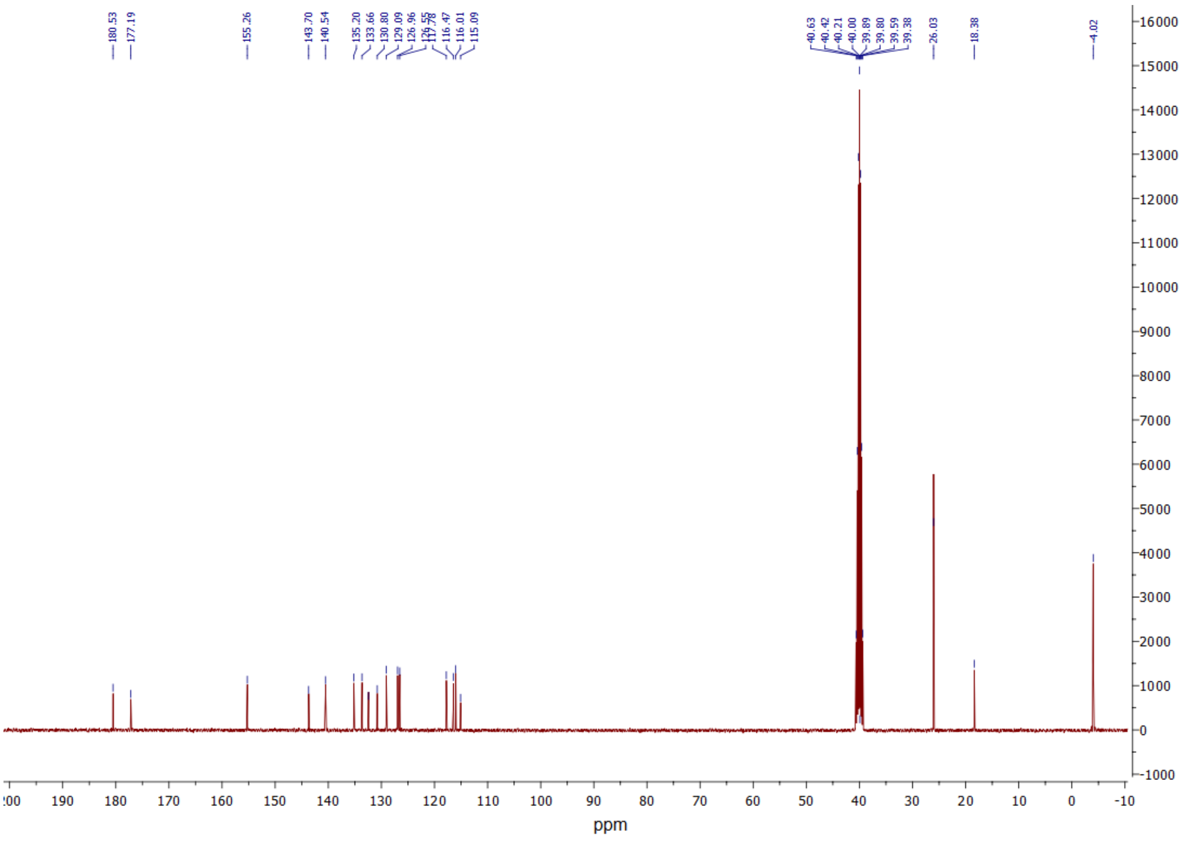

Figure S5. ^{13}C NMR spectrum of compound **NQ2a** (100 MHz, $CDCl_3$)

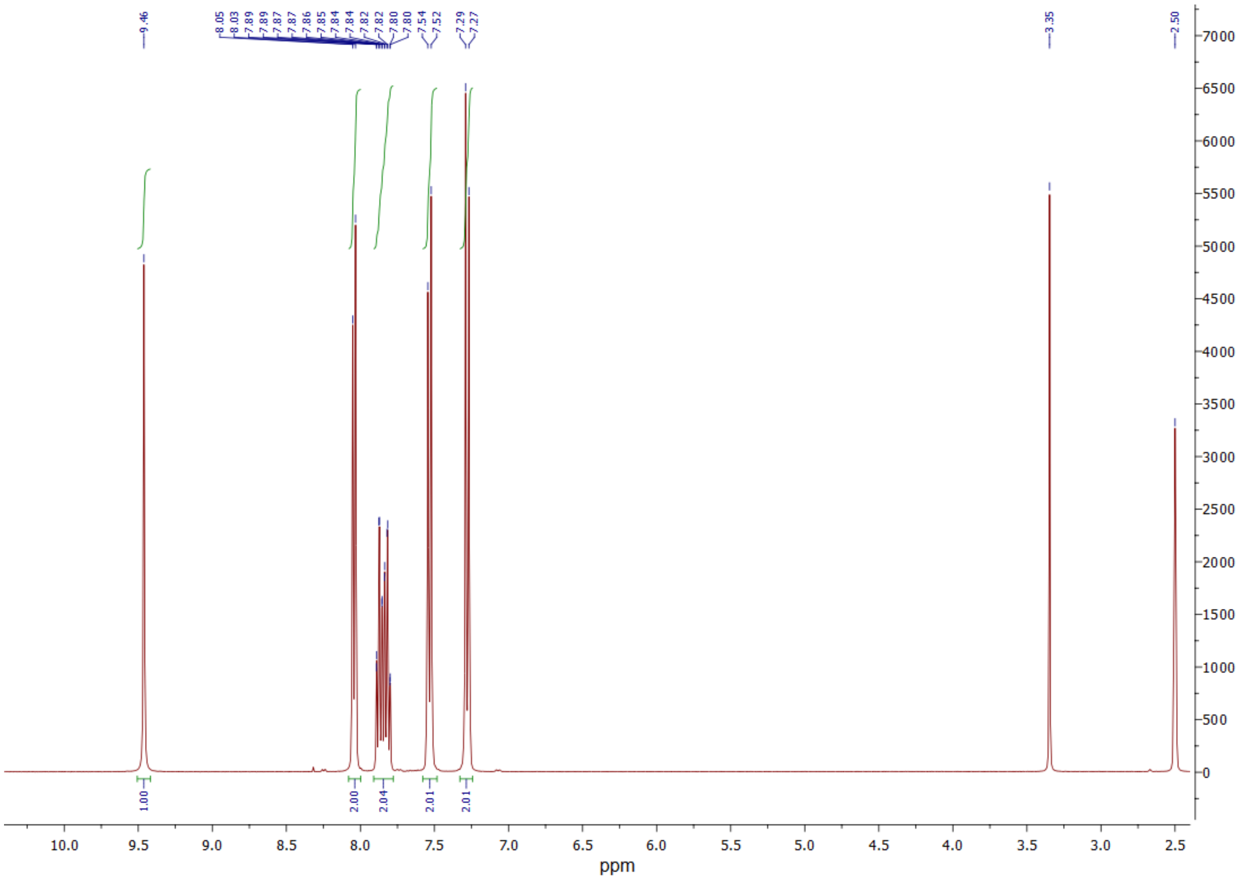

Figure S6. ^{1}H NMR spectrum of compound **NQS** (400 MHz, DMSO-d6)

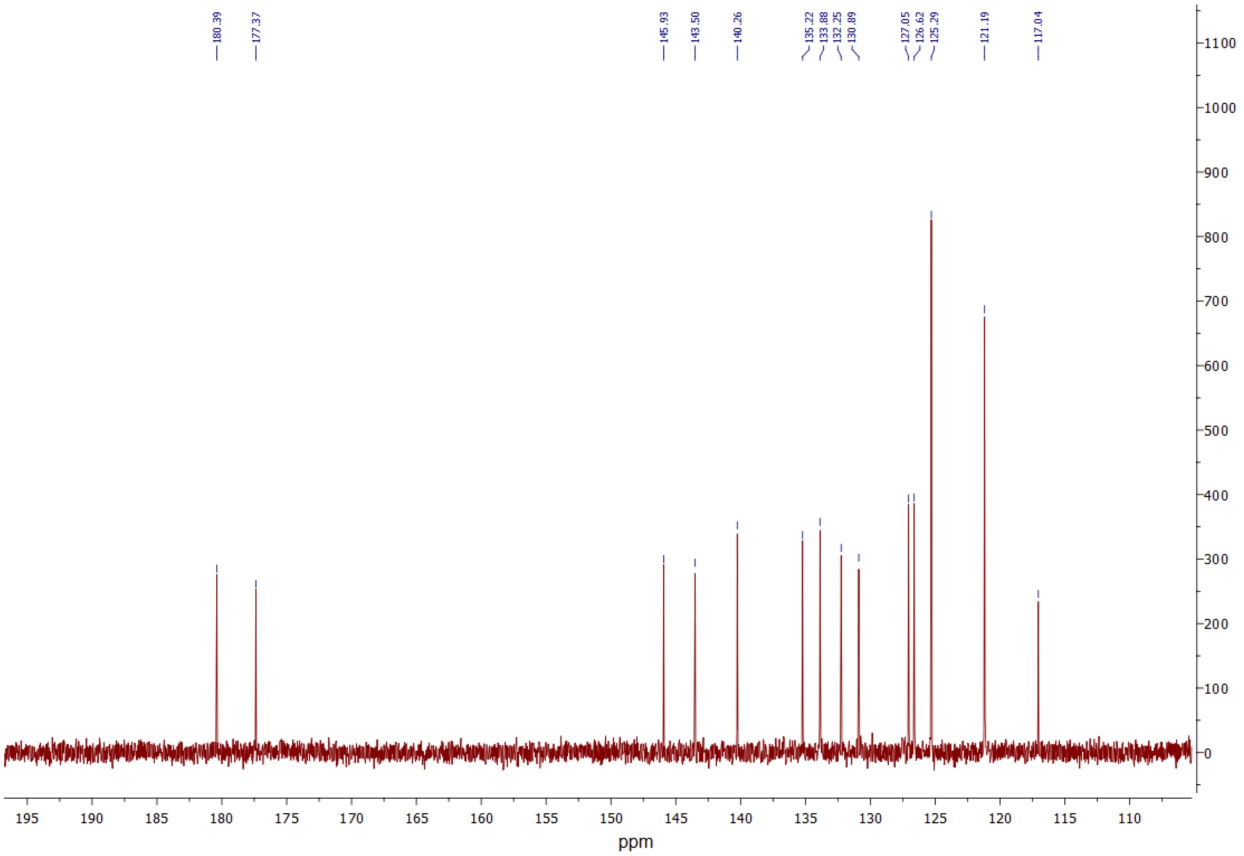

Figure S7. ^{13}C NMR spectrum of compound **NQS** (100 MHz, DMSO-d6)

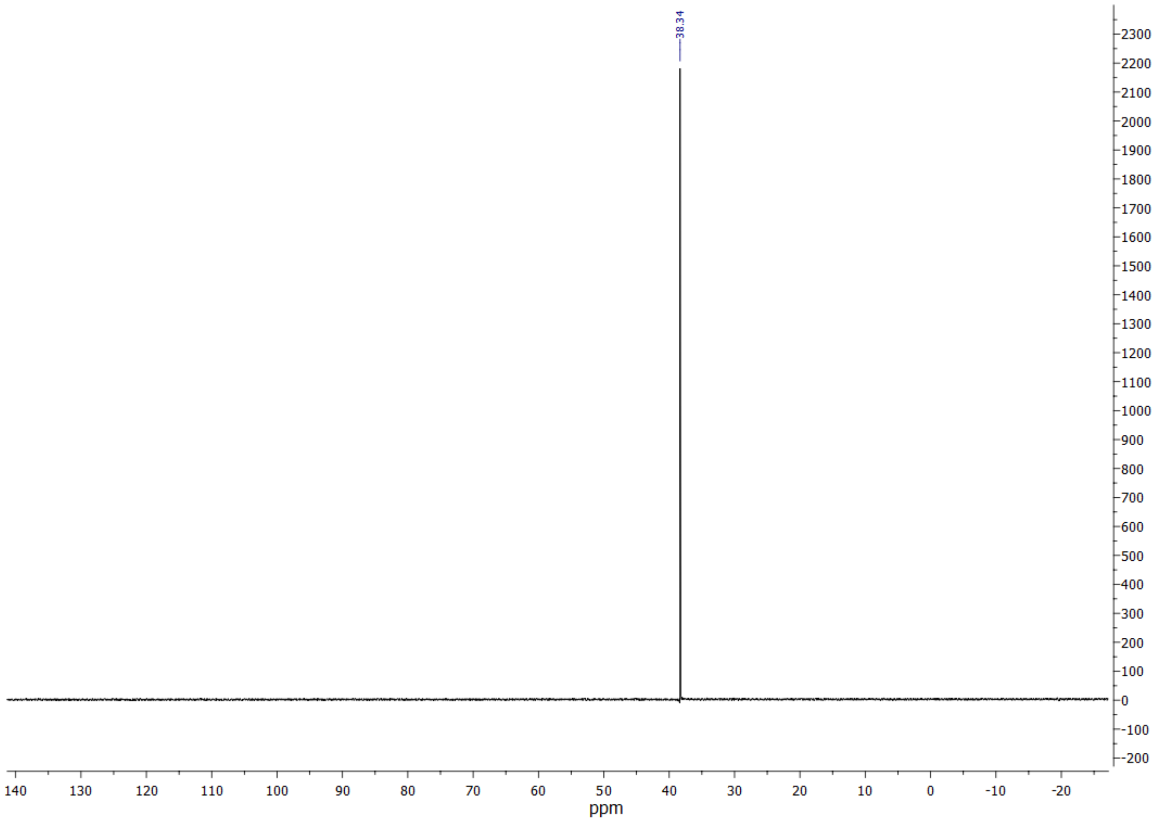

Figure S8. ^{19}F NMR spectrum of compound **NQS** (376 MHz, DMSO-d_6)

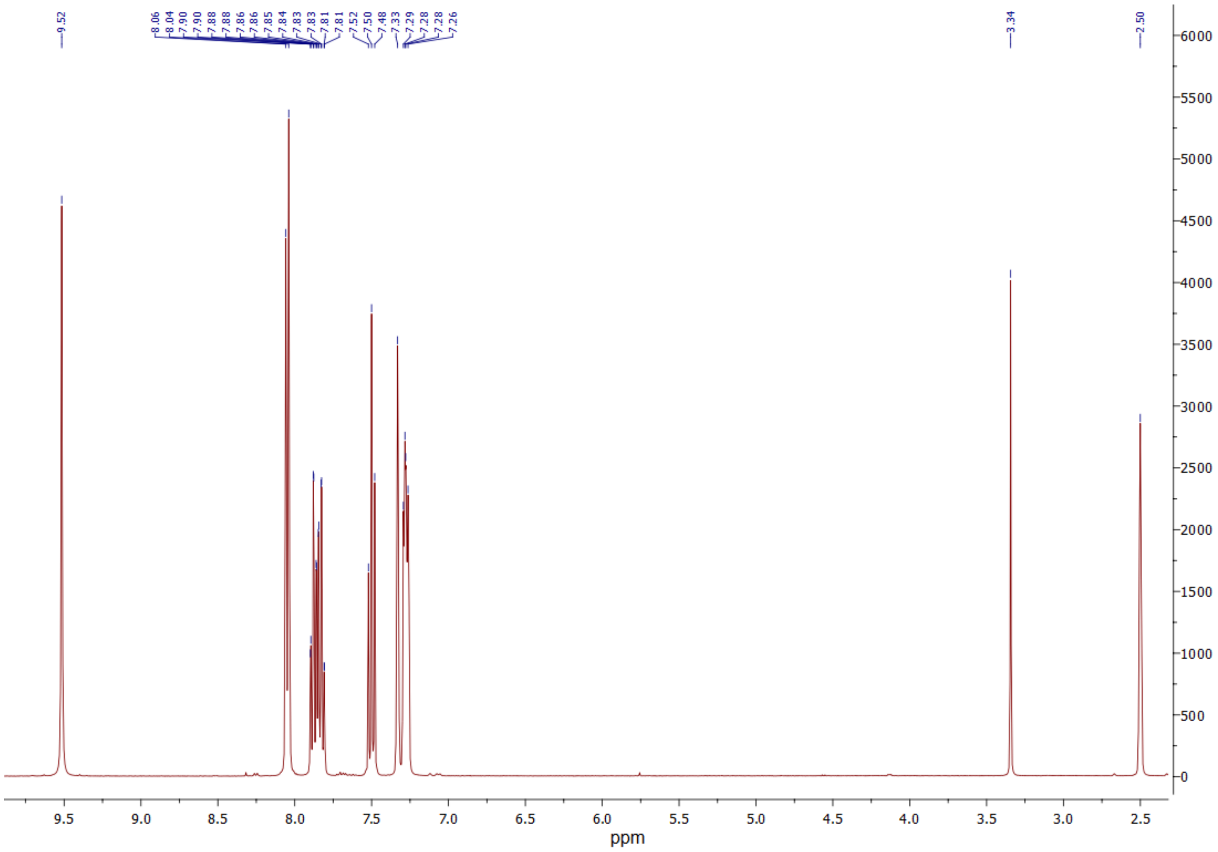

Figure S9. ^{1}H NMR spectrum of compound **NQS2** (400 MHz, DMSO-d_6)

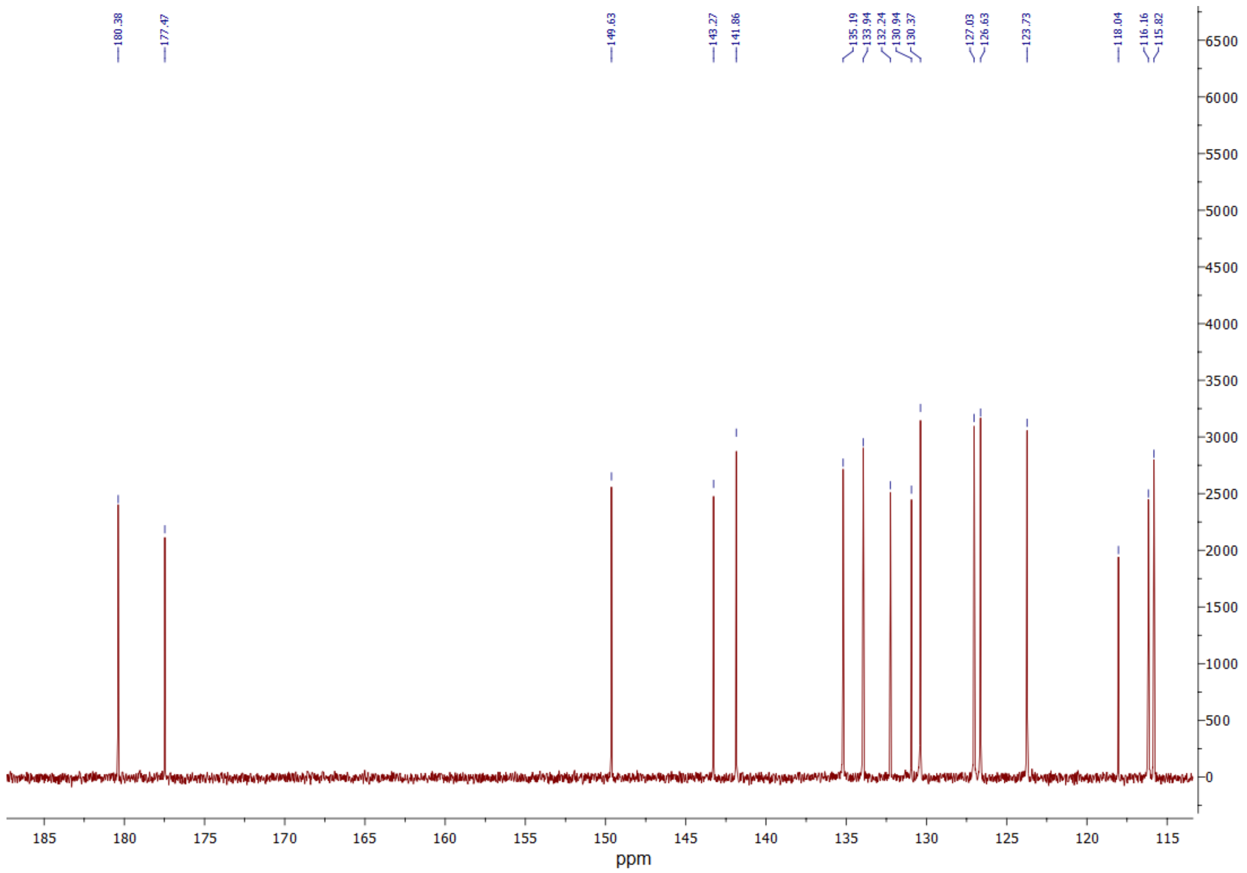

Figure S10. ^{13}C NMR spectrum of compound **NQS2** (100 MHz, DMSO-d_6)

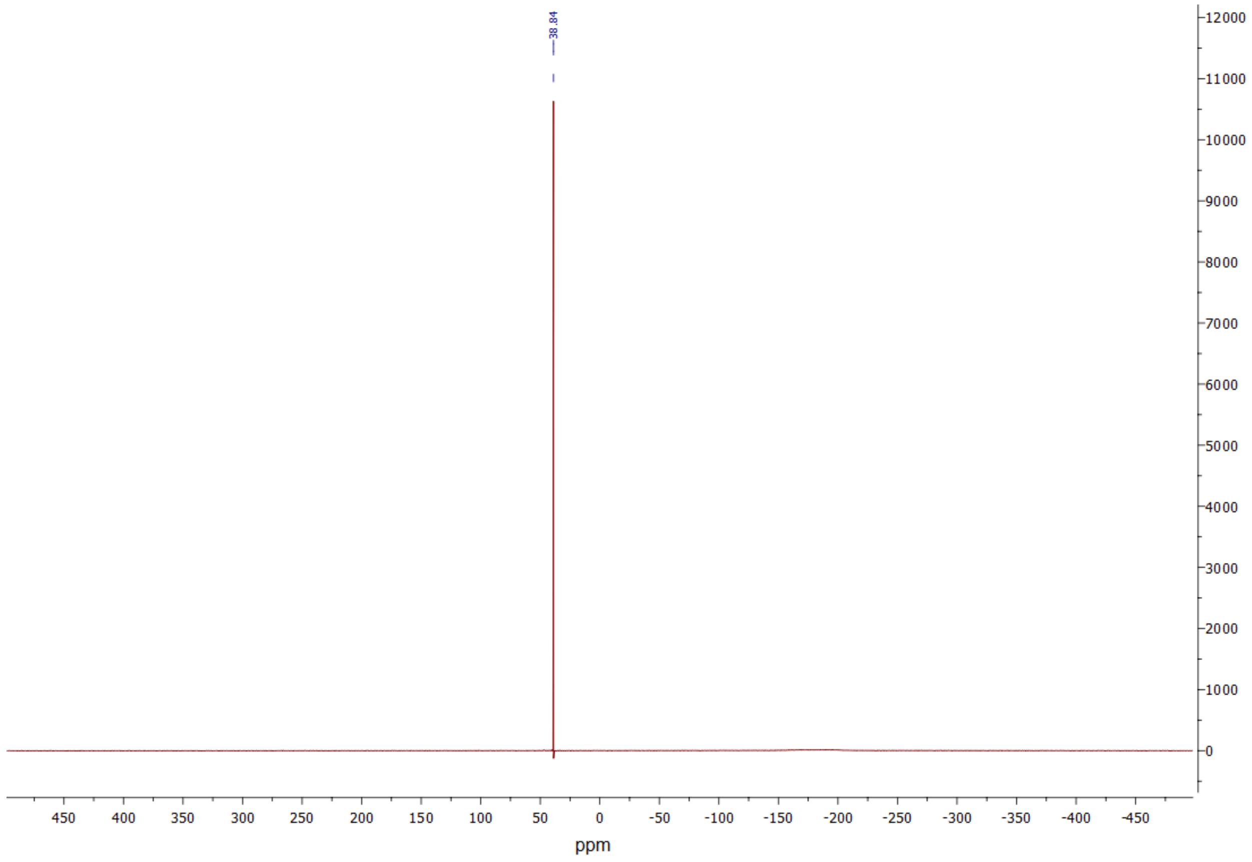

Figure S11. ^{19}F NMR spectrum of compound **NQS2** (376 MHz, DMSO-d_6)

Table S1. PASS prediction of biological activities for the naphthoquinone derivatives

Compound **NQ2**			Compound **NQ1**		
P_a	P_i	Activity	P_a	P_i	Activity
0.851	0.007	Antineoplastic	0,853	0,006	Aldehyde oxidase inhibitor
0.824	0.008	Aldehyde oxidase inhibitor	0,850	0,007	Antineoplastic
0.812	0.011	CYP2J2 substrate	0,789	0,015	CYP2J2 substrate
0.812	0.018	CYP2J substrate	0,792	0,023	CYP2J substrate
0.808	0.018	Antiseborrheic	0,778	0,023	Antiseborrheic
0.792	0.021	Chlordecone redustase inhibitor	0,765	0,027	Chlordecone reductase inhibitor
0.771	0.015	Membrane permeability inhibitor	0,745	0,022	Membrane permeability inhibitor
0.778	0.040	Ubiquinol-cytochrome-c redustase inhibitor	0,747	0,051	Ubiquinol-cytochrome-c reductase inhibitor
0.742	0.008	3-Hydroxybenzoate 6-monooxygenase inhibitor	0,703	0,011	3-Hydroxybenzoate 6-monooxygenase inhibitor
0.724	0.004	Indanol dehydrogenase inhibitor	0,722	0,045	Gluconate 2-dehydrogenase (acceptor) inhibitor
Compound **NQS2**			Compound **NQS**		
P_a	P_i	Activity	P_a	P_i	Activity
0,559	0,003	Antineoplastic (gastric cancer)	0.815	0.010	Antineoplastic
0,529	0,115	CYP2J substrate	0.738	0.038	CYP2J substrate
0,523	0,094	CYP2J2 substrate	0.724	0.027	CYP2J2 substrate
0,514	0,190	Gluconate 2-dehydrogenase (acceptor) inhibitor	0.689	0.012	3-Hydroxybenzoate 6-monooxygenase inhibitor
0,510	0,001	Elastase 2 inhibitor	0.651	0.004	Antineoplastic (carcinoma)
0,500	0,021	Antihypertensive	0.644	0.003	Antineoplastic (sarcoma)
0,559	0,003	Antineoplastic (gastric cancer)	0.634	0.003	Antineoplastic (gastric canser)
			0.683	0.066	Gluconate 2-dehydrogenase (acceptor) inhibitor
			0.633	0.031	Complement factor D inhibitor
			0.537	0.002	CDC25B inhibitor
Compound **NQ**					
P_a	P_i	Activity	P_a	P_i	Activity
0,908	0,003	Complement factor D inhibitor	0,870	0,008	CYP2J substrate
0,909	0,004	Chlordecone reductase inhibitor	0,869	0,005	Gluconate 2-dehydrogenase (acceptor) inhibitor
0,900	0,003	Arylsulfate sulfotransferase inhibitor	0,868	0,003	3-Hydroxybenzoate 6-monooxygenase inhibitor
0,894	0,006	Phobic disorders treatment	0,867	0,004	Cl--transporting ATPase inhibitor
0,888	0,010	Aspulvinone dimethylallyltransferase inhibitor	0,866	0,020	CYP2C12 substrate
0,886	0,005	Antiseborrheic	0,866	0,020	Membrane integrity agonist
0,880	0,002	Antineoplastic (small cell lung cancer)	0,865	0,004	DNA-(apurinic or apyrimidinic site) lyase inhibitor
0,876	0,004	2-Hydroxymuconate-semialdehyde hydrolase inhibitor	0,861	0,006	CYP2J2 substrate
0,876	0,004	Glycosylphosphatidylinositol phospholipase D inhibitor	0,857	0,004	Alkane 1-monooxygenase inhibitor
0,874	0,010	Ubiquinol-cytochrome-c reductase inhibitor	0,855	0,004	IgA-specific serine endopeptidase inhibitor
0,908	0,003	Complement factor D inhibitor	0,851	0,006	NADPH peroxidase inhibitor

Table S2. The electronic structure data

Compound	E(HOMO), eV	E(LUMO), eV	VEA, kcal/mol	η, eV	χ, eV	ω, eV
**NQ**	-9.858	-1.914	49.882	3.972	5.886	4.361
**NQ1**	-8.001	-1.476	42.156	3.262	4.738	3.441
**NQ2**	-8.291	-1.524	43.247	3.383	4.907	3.559
**NQS**	-8.635	-1.762	48.932	3.436	5.198	3.932
**NQS2**	-8.616	-1.639	46.197	3.488	5.127	3.768
